# Developmental Patterning as a Quantitative Trait: Genetic Modulation of the Hoxb6 Mutant Skeletal Phenotype

**DOI:** 10.1371/journal.pone.0146019

**Published:** 2016-01-22

**Authors:** Claudia Kappen

**Affiliations:** Department of Developmental Biology, Pennington Biomedical Research Center/Louisiana State University System, 6400 Perkins Road, Baton Rouge, Louisiana, 70808, United States of America; Instituto Gulbenkian de Ciência, PORTUGAL

## Abstract

The process of patterning along the anterior-posterior axis in vertebrates is highly conserved. The function of Hox genes in the axis patterning process is particularly well documented for bone development in the vertebral column and the limbs. We here show that Hoxb6, in skeletal elements at the cervico-thoracic junction, controls multiple independent aspects of skeletal pattern, implicating discrete developmental pathways as substrates for this transcription factor. In addition, we demonstrate that Hoxb6 function is subject to modulation by genetic factors. These results establish Hox-controlled skeletal pattern as a quantitative trait modulated by gene-gene interactions, and provide evidence that distinct modifiers influence the function of conserved developmental genes in fundamental patterning processes.

## Introduction

Hox genes are well known for their role in embryonic patterning in vertebrates [1], particularly in the skeleton, which provides the major structural component of body plans. Numerous investigations have shown that individual Hox genes control shape and identity of skeletal elements in region-specific fashion, with functional overlap of several Hox genes in each body region. These studies have advanced a model where the collective action of Hox genes during early development determines the shape of structures in the future skeleton.

This process of skeletal patterning itself is highly conserved, producing the stereotypical skeletal plan for each species. However, variable expressivity and penetrance were noted for some phenotypes in mice with Hox gene mutations [2–5]. For example, in a targeted mutant for the entire Hoxb6 gene, Rancourt et al. found that only a fraction of the animals displayed all the skeletal anomalies that collectively constitute the mutant phenotype, and that in some mice, alterations occurred unilaterally [5]. It was noted before for Hoxb4 mutants [4], that genetic background may influence the manifestation of mutant phenotypes. In those mutants, defective closure of the sternum was variable in a hybrid genetic background but fully penetrant when the mutation was carried in the inbred 129SvEv strain. However, detailed studies on the nature of genetic modulation of skeletal patterning by strain background have not been performed to date.

Using mice with a targeted deletion of the homeobox in the Hoxb6 gene [6], we here provide evidence that the patterns of skeletal features generated by Hox genes are modulated by factors in the genetic background. The developmental plasticity in skeletal pattern revealed by our results indicates that specification of skeletal features conforms to a threshold model rather than an instructive program. Thus -despite high evolutionary conservation of its molecular constituents- Hox gene controlled developmental patterning behaves as a quantitative trait.

## Methods

### Hoxb6^hd^ mutant mice

The generation of the Hoxb6^hd^ mutant allele has been described [6]; the official designation is Hoxb6^tm1Cka^ (http://www.informatics.jax.org/allele/MGI:3514269). Briefly, the neo-cassette from pMC1POLA [7] was inserted as a blunted XhoI-SalI fragment into the Hoxb6 locus that was digested with ApaI and Bal31, deleting from the second exon 375 base pairs that encode the homeodomain and three amino acids N-terminal to it. Recombinant E14 ES cells were identified at a frequency of 1/60 by Southern blot and PCR [6] and used to construct E14->C57BL/6 chimeras. After germline transmission, intercrosses of progeny established that heterozygotes and homozygotes for the mutant allele are viable and fertile. Crosses of the mutant allele onto the C57BL/6 background were performed by backcrossing males heterozygous for the Hoxb6^hd^ mutant allele to C57BL/6 females obtained from The Jackson Laboratories (Bar Harbor, ME). Offspring were screened for the presence of wildtype and mutant loci by PCR using allele-specific primer sets as described [6]. Eight successive generations of backcrosses to C67BL/6 (G_1_-G_8_) were performed (details below), after which intercrosses in brother x sister matings (G_8_i) were set up to produce offspring homozygous for the Hoxb6^hd^ mutation. To cross the mutant allele into the 129Sv background, we obtained 129S6/SvEvTac mice from Taconic Farms (Taconic, NY). Males from the C57BL/6-Hoxb6^hd^ line heterozygous for the mutation were crossed to wildtype 129S6/SvEvTac females, and offspring (H_1_) were either used in brother x sister matings (H_1_i) or for further backcrosses (H_2_, H_3_) to 129S6/SvEvTac. Homozygotes for the Hoxb6^hd^ mutant allele on predominantly 129S6/SvEvTac background were generated from brother x sister matings of H_3_ offspring. Genotype was ascertained by PCR and homozygosity was diagnosed by presence of PCR product from the mutant and concomitant absence of amplification products from the wildtype locus. To control for potential effects of bedding conditions on phenotype, one group of mutant animals was maintained on non-foodstuff synthetic fiber bedding (Irradiated Isopads, Harlan, Indianapolis, IN). There were no significant differences in litter size between any of the experimental groups (S1 Table). All other animals were housed in individually vented microisolator cages on irradiated conventional bedding with ad libitum access to acidified water and food.

Studies reported in this manuscript were carried out in strict accordance with the recommendations in the Guide for the Care and Use of Laboratory Animals of the National Institutes of Health of the United States of America, covered by protocols approved by the Institutional Animal Care and Use Committees of the Mayo Clinic in Scottsdale, AZ, the University of Nebraska Medical Center in Omaha, NE, and the Pennington Biomedical Research Center in Baton Rouge, LA, respectively. The currently active protocol #828 (approved at Pennington Biomedical Research Center) is approved through January 28th 2016.

### PCR Conditions

Primers for the wildtype Hoxb6 locus were: forward primer: 5’-CGTAACAGGTTCCTCTT-3”; and reverse primer: 5’-CTTTCCTCCTCCTCCTCC-3’ with a product size of 250 bp; and primers for the mutant locus were: forward primer: 5’-AACCCCGCCCAGCGTCTTAT-3’ (in the pMC1POLA cassette, [7] and reverse primer: 5’-AAAGCACGAGGAAGCGGTCAG-3’, producing an amplicon of 850 bp size. PCR conditions for detection of the wildtype allele were: one cycle of 5 min. at 94°C, 1 min. at 52°C, 1 min. at 72°C, followed by 34 cycles of 30 sec. at 94°C, 1 min. at 52°C, 1 min. at 72°C, followed by a final cycle of 30 sec. at 94°C, 1 min. at 52°C, 5 min. at 72°C and hold at 4°C. For detection of the mutant allele, the same conditions were used except that the annealing temperature was 58°C. PCR products were resolved on 1% agarose gels by EtBr staining and sizes were determined relative to 1kb ladder (Gibco/Invitrogen).

### Preparation and staining of skeletons

Newborn mouse skeleton preparations and staining for Alizarin Red (bone) and Alcian Blue (cartilage) were carried out as described [8]. Adult skeletons were prepared by employing dermestid beetles (Carolina Biological Supply Company) and subsequent clearing in 1% KOH under agitation for at least one month. After inspection and photography (Nikon F3) as whole-mount specimen, skeletons were dissected into individual vertebrae counting from the base of the skull. Scoring of features was done independently by two individuals on a Leica M6 Stereomicroscope, and photography was performed using a Kodak MDS 290 digital camera.

### Radiology of adult mice

X-ray examinations on adult mice from backcrosses were conducted using a Faxitron CM50 (IN/US Systems, Tampa FL). Briefly, animals were anesthetized with Avertin (15 μl/gr body weight of a 2.5% solution) and digital X-ray images were taken in frontal and lateral views. Scoring of features was performed independently by two individuals.

### Whole-mount staining and in situ hybridization of embryos

Staining of mouse embryos isolated at gestational day 13.5 for Alcian Blue was performed as described previously [9].

In situ hybridization for Hoxb6 in embryos was performed on whole-mount specimen following the protocol of Rosen and Beddington as published [10], except for several minor modifications: A proteinase K treatment step was performed for embryos older than E9.5, prehybridization and hybridization incubations were performed at 65°C, and the blocking step, antibody incubation, washes, and detection of enzymatic activity steps contained 2 mM levamisole. In situ hybridization to sections from paraffin-embedded embryos was done as previously published [8].

### Statistical evaluation

Statistical evaluation of results was performed by 2-tailed Fisher’s exact test (http://www.matforsk.no/ola/fisher.htm). Litter averages and standard deviations were calculated as arithmetic means. Associations of features were analyzed by linear regression, significance of relationships was determined using ANOVA, and variance was estimated using the VAR/VARP function in Microsoft Excel. Adjustment for multiple testing was done by Bonferroni correction. Relative risk and confidence intervals were calculated as implemented on http://www.hutchon.net/calcmenu.htm.

## Results

### Rib abnormalities in Hoxb6 mutants

**Fig 1:**
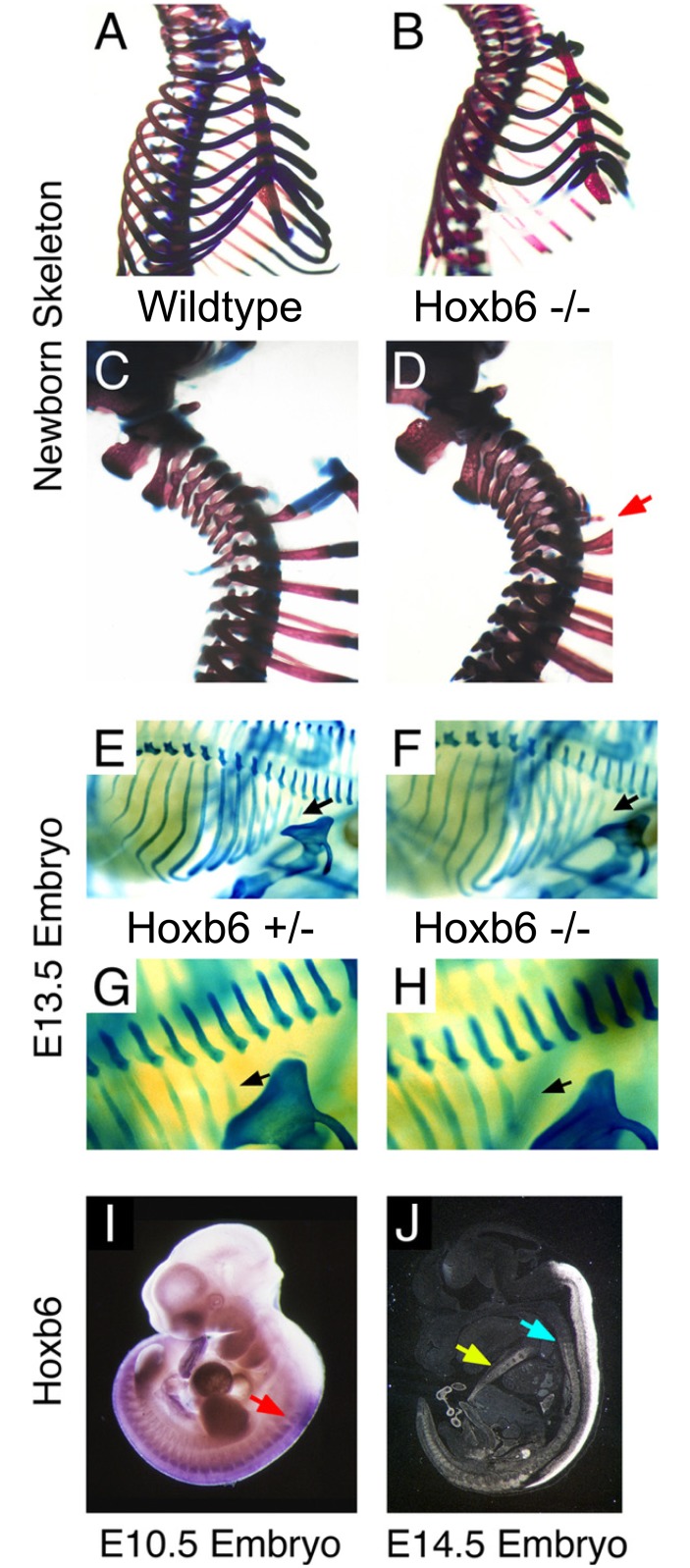
Hoxb6 mutants exhibit skeletal alterations and absence of the first pair of ribs. Panel A: Rib cage of wildtype skeleton with 7 ribs attached to the sternum and 6 sternebrae. Panel B: Rib cage of mutant with 6 ribs attached to sternum and 5 sternebrae. Panel C: Cervico-thoracic region of wildtype newborn skeleton. Panel D: Cervico-thoracic region of Hoxb6^hd^ mutant newborn skeleton, the red arrow points to small ossified structures in place of the first pair of ribs. Panel E: E13.5 embryo heterozygous for the Hoxb6^hd^ mutant allele stained with Alcian Blue reveals normal cartilage anlagen. Panel F: E13.5 homozygous Hoxb6^hd^ mutant embryo with cartilage anlagen for the first rib absent (compare targets of black arrows). Panels G, H: Magnifications of Panels E and F, respectively. Panel I: Whole mount in situ hybridization for Hoxb6 in an embryo isolated at day E10.5. The anterior limit of Hoxb6 expression in somites is found within the caudal region of the somite that contributes to prevertebra 6 (red arrow). Panel J: In situ hybridization to a sagittal section from an embryo at E14.5 shows strong signal in spinal cord, and Hoxb6 expression is evident in the vertebral column (light blue arrow), and in the precursors to the sternum (yellow arrow).

Mice homozygous for a targeted deletion of the homeobox in the Hoxb6 gene (Hoxb6^hd^) typically have only six pairs of ribs attached to the sternum (Fig 1, Panels A, B) and eight cervical-like vertebrae (Fig 1, Panels C, D). Absence of ribs is caused by lack of formation of the cartilage anlagen for the first pair of ribs in Hoxb6^hd^ mutant embryos (Fig 1, Panels E-H). This phenotype is consistent with an earlier report on a targeted mutation in the first exon of the Hoxb6 gene (Hoxb6^ex1^, [5]), and with the expression of Hoxb6 in the developing embryo (Fig 1 Panels I, J). Hoxb6 mRNA is detected in neural tube and somites, with an anterior boundary in somites that contribute to the sixth prevertebra. Consistent with this anterior limit of expression, phenotypic alterations in Hoxb6^hd^ mutants are present in and posterior to the sixth cervical vertebra (skeletal element C6).

**Fig 2:**
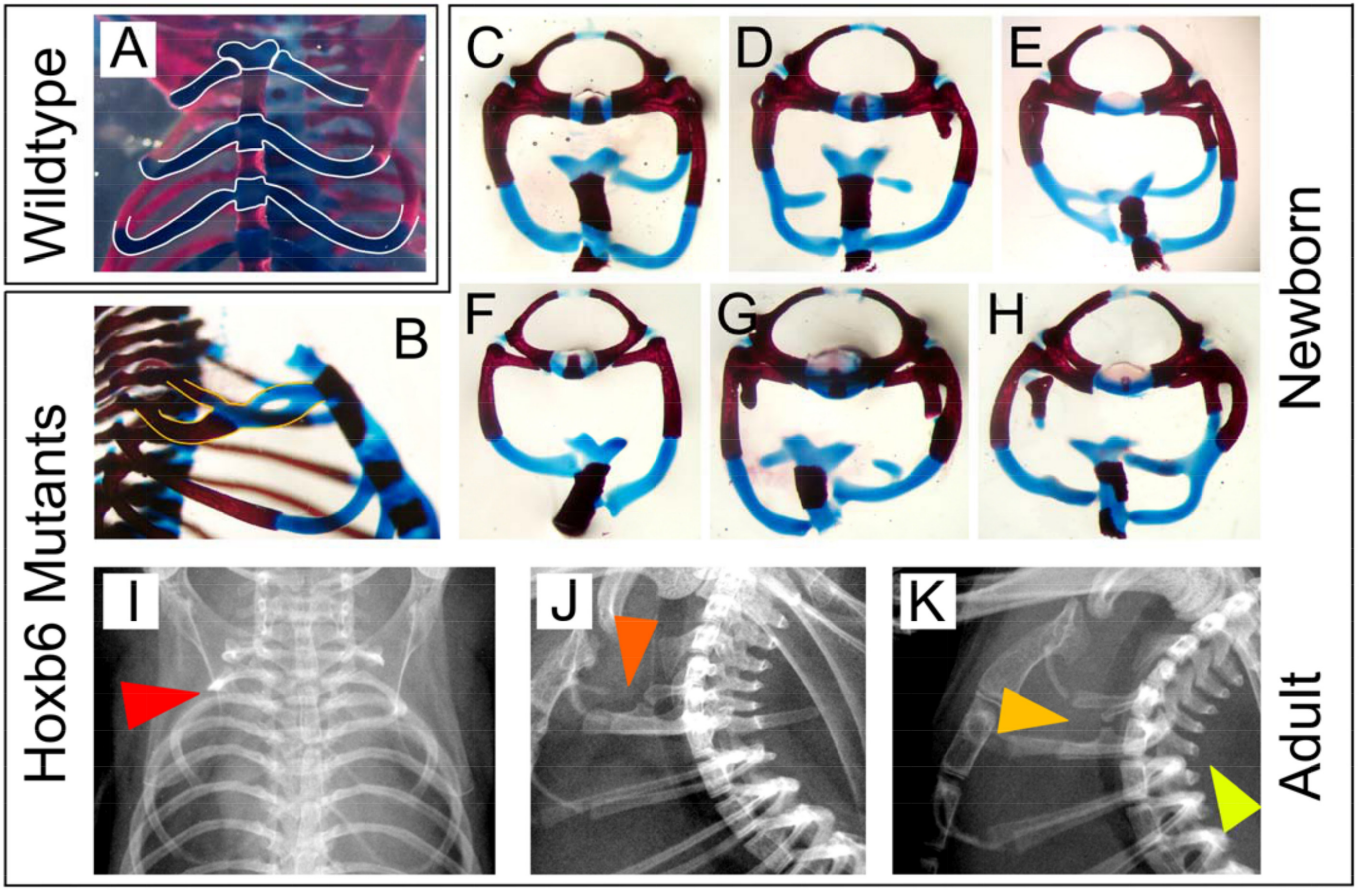
Features of rib development and sternal articulation in Hoxb6^hd^ mutants. Panel A: Newborn wildtype skeleton with outlines (white) of cartilaginous portions of the ribs and sternum. Panels B-K: Hoxb6^hd^ mutant skeletons from newborns (B-H) or adults (I-K). Panel B: Note the crossover of rib from the eighth vertebra and fusion with the rib from ninth vertebra, bifurcation of cartilage, and aberrant attachment of fused cartilage, and fusion of the first two sternebrae. Panels C-H: Different combinations in individuals of absent or rudimentary ribs, unilaterally (C-E) or bilaterally (F-H), defective formation of rib cartilage (D, E, G, H), crossovers (D, E, H), fusions (E, H), bifurcations (D, E, H), aberrant articulation to the sternum (C-H) and off-set sternal attachment of the ribs (H). All preparations contain the eighth and ninth vertebrae, except in Panel F, which represents the ninth vertebra. Panels I-K: X-rays of individual adult Hoxb6^hd^ mutants. Panel I: frontal view; note truncation of first rib unilaterally (red arrow). Panel J: lateral view, orange arrow points to crossover and defective sternal rib cartilage. Panel K: lateral view, dark yellow arrow points to crossover and unilateral absence of first rib, bright yellow arrow points to absence of spinous process on the ninth vertebra.

The most obvious feature of the Hoxb6^hd^ phenotype at the gross level is the absence or maldevelopment of ribs normally associated with the first thoracic (eighth) vertebra. Fig 2 depicts the most profound defects in rib development observed in Hoxb6^hd^ mutants, including rib truncations, bifurcations, fusions, and aberrant attachment of the cartilaginous portion of the ribs to the sternum. Rib abnormalities carry into adulthood and are detectable on X-rays (Fig 2, Panels I-K). Taken together, these results implicate Hoxb6 in patterning of skeletal elements in the region of the cervico-thoracic junction, and in rib cartilage formation.

### Homeotic transformation in Hoxb6 mutants

**Fig 3:**
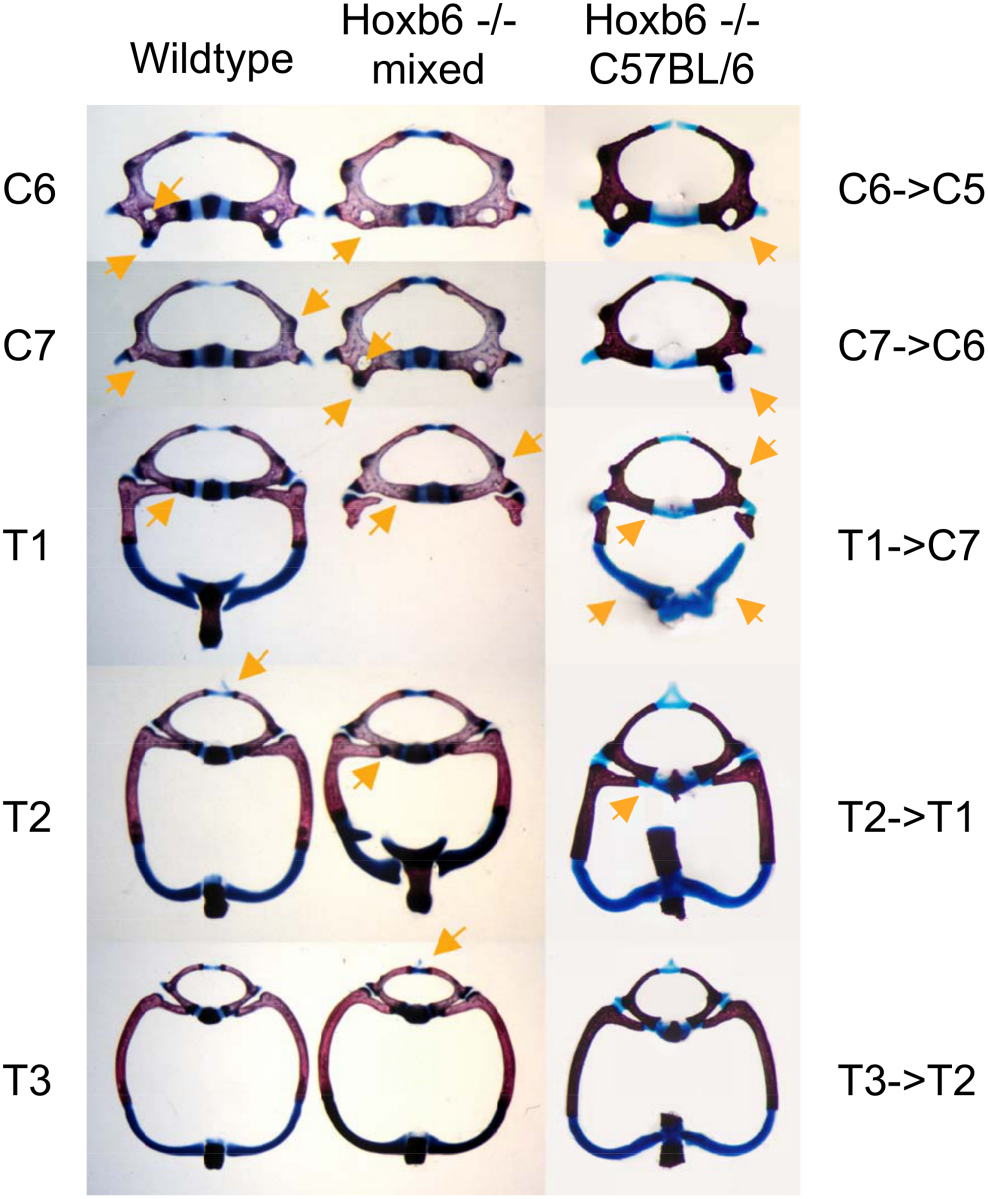
Phenotype expressivity in Hoxb6 mutants on different genetic backgrounds. Panels in column A: Vertebral elements of wildtype newborn mouse skeleton. Arrows point to characteristic features: Vertebra C6: vertebral foramen and anterior tuberculum; C7: lateral extension of the vertebral body and absence of foramen; T1: articulation of rib capitulum to vertebral body; T2: dorsal cartilage extension (processus spinosus). Panels in column B: Homeotic transformations in a homozygous Hoxb6^hd^ mutant on mixed background. Arrows point to features found transposed to vertebrae at the next axial level, resembling anterior homeotic transformations of these skeletal elements. Panels in column C: Homeotic transformations in homozygous Hoxb6^hd^ mutant on C57BL/6 background. Homeotic transformations are more often found unilaterally; arrow points to rib cartilage associated with the transformed eighth vertebra.

In a detailed study, individual skeletal elements were examined for the presence of the following characters: "open foramen in C5", "transformation C6->C5", "transformation C7->C6", "transformation T1->C7", "articulation of ribs to eighth vertebra", "vertebral rib development", "sternal rib development", "articulation of distal ribs to sternum", "position of spinous process". These analyses revealed changes in shape of vertebrae that resemble anterior homeotic transformations (Fig 3, Panel B) of vertebrae 6 through 10 (C6->C5, C7->C6, T1->C7, T2->T1, T3->T2). In addition, we found variations in extent of closure and ossification of the vertebral foramina of C5; such open foramina also occur in wildtype and heterozygous mutants, indicating that they are not related to mutation of the Hoxb6 gene.

### Manifestation of abnormalities in Hoxb6 mutants is dependent on strain genetic background

After generating a congenic strain with the Hoxb6^hd^ mutant allele on the C57BL/6 genetic background, we noticed that those mutants generally had seven rib pairs articulating to the sternum, in contrast to our earlier observations for mutants that had been maintained in a mixed genetic background of C57BL/6 and 129S6/Sv. In the Hoxb6^hd^ congenic strain on the C57BL/6 background, the first rib pair was usually present, although some abnormalities were found (Fig 3, Panel C). Most notably, the rib heads that formed in the region of the eighth vertebra were shorter than normal and often lacked the rib head (capitulum) and proper articulation to the vertebral body. Articulation to the sternum was present and, except for a few cases, looked normal. Detailed analyses revealed that C57BL/6-Hoxb6^hd^ homozygotes still develop transformations of vertebrae C6-T3, as judged by vertebral shape (Fig 3). Thus, the Hoxb6^hd^ mutation on the C57BL/6 inbred background affects the same vertebral elements as in the mixed genetic background, causing homeotic transformations.

**Fig 4:**
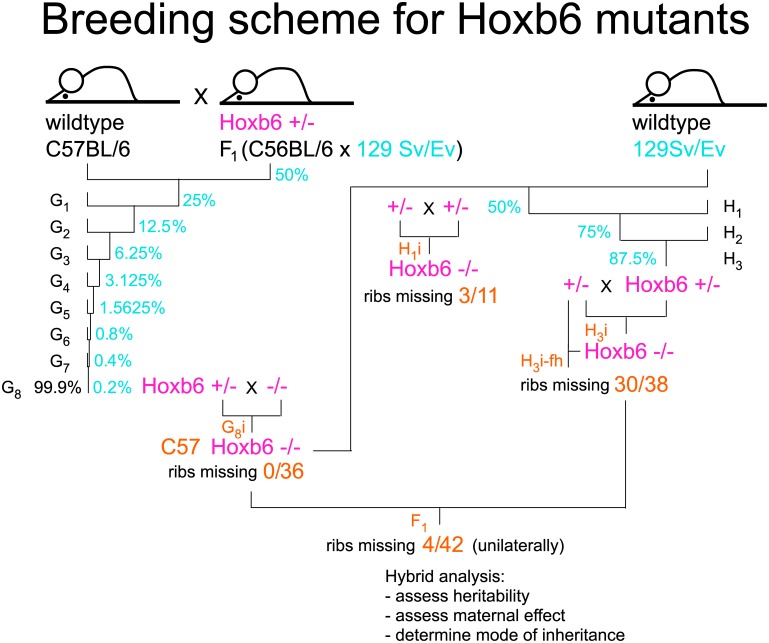
Breeding scheme for C57BL/6-Hoxb6^hd^ congenic strain and backcross to 129S6/SvEv. A male heterozygote for the Hoxb6^hd^ mutation was crossed to C57BL/6 wildtype females and male offspring were used for further backcrosses. For the backcross to 129S6/SvEv wildtype, a Hoxb6^hd^ homozygous male was used, and offspring from this cross (H_1_) were intercrossed (H_1_i) to generate mixed background animals homozygous for the Hoxb6 mutation. Out of 11 H_1_i progeny, 3 had defective first rib development, indicating an effect of the 129S6/SvEv genetic background on phenotype manifestation. Further backcrosses used H_1_ males heterozygous for the Hoxb6^hd^ mutation and wildtype 129S6/SvEv females. Intercrosses of H_3_ animals (H_3_i) yielded homozygous Hoxb6^hd^ mutants on predominantly 129S6/SvEv genetic background. To exclude a possible developmental disadvantage for the 25% homozygotes in a cross of Hoxb6^hd^ heterozygous parents, we set up crosses between H_3_i generation animals in which the father was homozygous for the Hoxb6^hd^ mutation (H_3_i-fh), thus increasing the yield of homozygous mutants to 50%, providing equal chance for intrauterine development. Of 38 mutant H_3_i progeny, 33 exhibited the “missing rib” phenotype, confirming the influence of genetic background on phenotype manifestation in Hoxb6^hd^ mutants. Further backcrosses used homozygous mutant H_1_i progeny bred to C57BL/6-Hoxb6^hd^ congenics (G_8_i), which produced 42 K_2_ progeny, of which 12 exhibited defective ribs; crosses of Hoxb6^hd^ homozygous mutant F_1_ hybrids to H_3_ Hoxb6^hd^ homozygous mutants produced 62 J_2_ animals, of which 55 had defective ribs. Differences in incidence of the phenotype are statistically significant between all groups (p = or < 0.01), except for the J_2_/H_3_ comparison. These results provide evidence that genetic background controls rib development in Hoxb6^hd^ mutants in intermediate fashion.

However, the severity of the Hoxb6^hd^ mutant phenotype in the rib cage was reduced in C57BL/6 compared to a background of mixed composition. Several explanations could explain this finding: (i) Different housing conditions during the initial studies and for the C57BL/6 congenic strain at the time of investigation could affect phenotype expressivity. (ii) Phenotype manifestation may be influenced by genetic composition of the mother, as inbred mouse strains vary in length of pregnancy, physical activity and metabolic regulation, all of which can affect embryonic development. (iii) The 129S6/Sv genetic background confers a modifier locus that increases phenotype severity. In order to systematically evaluate these possibilities, we analyzed the effects of the Hoxb6^hd^ mutation under different housing and genetic conditions. The breeding scheme for crosses on different genetic backgrounds is shown in Fig 4; no significant differences in litter sizes were found between groups of progeny (S1 Table). For all offspring, skeletons were prepared, and features of individual skeletal elements were scored according to the characters depicted in Fig 3.

**Fig 5:**
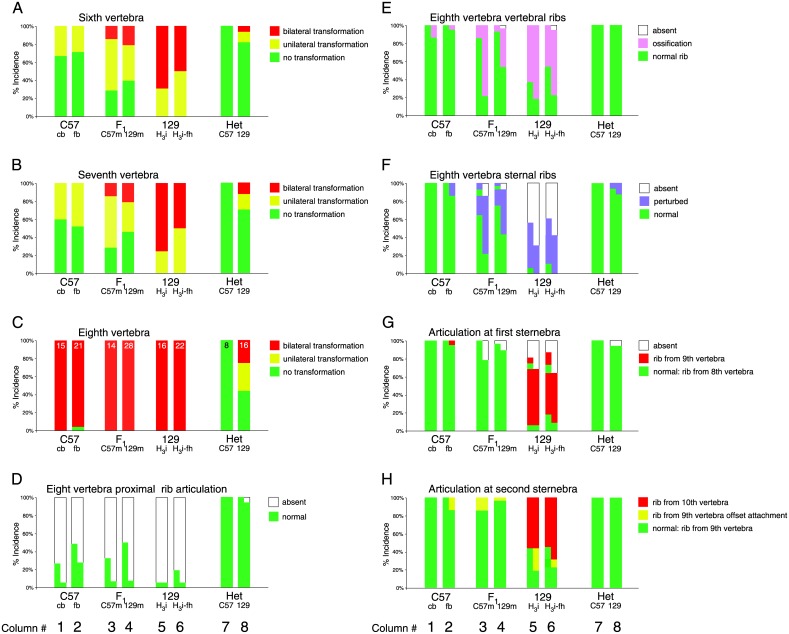
Phenotype expressivity in Hoxb6 mutants is controlled by genetic background of the embryo. Columns show the quantitative distribution of features by skeletal element and genetic background. Phenotype features were scored exactly as shown by orange arrows in Fig 3. Panel A: 6^th^ vertebra; Panel B: 7^th^ vertebra; Panel C: 8^th^ vertebra (normally first thoracic vertebra); Panel D: Articulation of ribs/ossifications to the 8^th^ vertebrae; Panel E: Proximal (vertebral) ribs; Panel F: Distal (sternal) ribs; Panel G: Articulation to sternum at level of 8^th^ vertebra (normally T1); Panel H: Articulation to sternum at level of 9^th^ vertebra (normally T2). For each experimental group, the fraction of animals with a given phenotype is plotted, and the total number of animals is given as a number in the respective columns of Panel C. No attempt was made to depict side of unilateral anomalies; as there was no preference, they were grouped together with increasing severity to the right of each column. Columns 1–8 correspond to the following groups: 1: Progeny from crosses of homozygous C57BL/6-Hoxb6^hd^ mutant males to heterozygous C57BL/6-Hoxb6^hd^ mutant females (G_8_i, see Fig 4) maintained on corn-cob bedding (C57 cb). 2: Progeny from crosses of homozygous C57BL/6-Hoxb6^hd^ mutant males to heterozygous C57BL/6-Hoxb6^hd^ mutant females (G_8_i) maintained on synthetic fiber bedding (C57 fb). 3: Progeny (F_1_) from crosses of homozygous H_3_ mutant males to homozygous C57BL/6-Hoxb6^hd^ females (C57m: maternal uterine environment is C57BL/6). 4: Progeny (F_1_) from crosses of homozygous C57BL/6-Hox-6^hd^ mutant males to homozygous H_3_ mutant females (129m: maternal uterine environment is predominantly 129Sv/Ev). 5: Progeny (H_3_i) from brother x sister matings of H_3_ generation animals on predominantly 129S6/SvEv background. 6: Progeny from crosses of homozygous H_3_ mutant males to heterozygous H_3_ mutant females (H_3_i-fh; homozygous father) on predominantly 129S6/SvEv background. 7: C57BL/6-Hoxb6^hd^ heterozygous mutants (Het C57). 8: Heterozygous Hoxb6^hd^ mutants from H_3_i intercrosses (Het 129). Statistical significance was established by 2-tailed Fisher’s exact test. For comparison of heterozygotes on the two different genetic backgrounds (Columns 7 and 8), p-values were not significant except for T1 status (p = 0.0095). For complete p-value matrices for all pairwise comparisons for columns 1–6 see Table 1.

Heterozygous C57BL/6-Hoxb6^hd^ females were maintained in two housing conditions: corncob bedding (which the mice also like to eat) and non-foodstuff synthetic fiber bedding. After mating to homozygous C57BL/6-Hoxb6^hd^ males, skeletons of progeny were compared. Fig 5 reveals (compare Columns 1 and 2) no significant differences for incidence or severity of vertebral transformations or rib abnormalities in homozygous mutants; heterozygotes also were indistinguishable between both conditions. Thus, housing conditions had no detectable effect on phenotype manifestation in Hoxb6^hd^ mutants.

To investigate maternal influence and genetic background as possible parameters, we crossed C57BL/6 Hoxb6^hd^ congenic animals to wildtype 129S6/SvEvTac mice. The resulting progeny is F_1_ hybrid (50%/50%) for genetic background (here called H_1_) and heterozygous for the Hoxb6^hd^ mutation. A further increase of 129S6/SvEv background was achieved by backcrossing H_1_ hybrid Hoxb6^hd^ heterozygotes to 129S6/SvEv for two more generations (see Fig 4), generating backcross generation 3 (H_3_) animals (with approximately 87.5% of their genomes of 129S6/SvEvTac origin). Brother-sister intercrosses (H_3_i) will produce homozygous mutants on a predominantly 129S6/SvEv background. It is important to note here that these crosses used females heterozygous for the mutant allele, so that potential effects of homozygosity at the Hoxb6 mutant locus on fertility and fecundity were excluded.

In Hoxb6^hd^ mutants on the 129S6/SvEv genetic background (Fig 5 Column 5), there was higher incidence of vertebral transformations and rib abnormalities, with increased occurrence on both sides of the animal (for significance values, see Table 1). These data indicate that the 129S6/SvEv genetic background promotes a more severe phenotype manifestation in Hoxb6^hd^ mutants. To exclude the possibility that the 25% homozygous mutants arising from a cross of heterozygous parents are simply at a developmental disadvantage relative to their littermates, and therefore more strongly affected, we also analyzed litters with equal chance for homozygotes and heterozygotes from a cross of homozygous Hoxb6^hd^ males to heterozygotes females (H_3_i-fh; father homozygous). There was no significant difference to the outcomes of the previous cross (Fig 5, compare Columns 5 and 6). Taken together, these results clearly show that the Hoxb6^hd^ mutation exhibits differential phenotype manifestation dependent on genetic background.

**Table 1:** Statistical evaluation of results in Fig 5 by phenotype feature.

Phenotype feature		Experimental groups (n = Number of individuals)					
#	Experimental group	n = 15	n = 21	n = 14	n = 28	n = 16	n = 22
**C6->C5 transformation**		C57 cb	C57 fb	F_1_:C57m	F_1_:129m	129 H_3_i	129 H_3_i-fh
1	C57 cb	-	NS	NS	NS	**6.8 x 10**^**−5**^	**8.6 x 10**^**−6**^
2	C57 fb		-	8.1 x 10^−3^	NS	**6.1 x 10**^**−6**^	**3.6 x 10**^**−7**^
3	F_1_:C57m			-	NS	NS	2.5 x 10^−2^
4	F_1_:129m				-	**3.0 x 10**^**−3**^	**1.1 x 10**^**−3**^
5	129 H3i					-	NS
6	129 H3i-fh						-
**C7->C6 transformation**		C57 cb	C57 fb	F_1_:C57m	F_1_:129m	129 H_3_i	129 H_3_i-fh
1	C57 cb	-	NS	NS	NS	**2.5 x 10**^**−4**^	**4.0 x 10**^**−5**^
2	C57 fb		-	NS	NS	**6.1 x 10**^**−4**^	**6.1 x 10**^**−5**^
3	F_1_:C57m			-	NS	3.7 x 10^−2^	1.7 x 10^−2^
4	F_1_:129m				-	**1.3 x 10**^**−3**^	**1.6 x 10**^**−4**^
5	129 H3i					-	NS
6	129 H3i-fh						-
**T1 vertebral ribs**		C57 cb	C57 fb	F_1_:C57m	F_1_:129m	129 H_3_i	129 H_3_i-fh
1	C57 cb	-	NS	**6.8 x 10**^**−4**^	4.4 x 10^−2^	**2.4 x 10**^**−4**^	**1.9 x 10**^**−4**^
2	C57 fb		-	**9.2 x 10**^**−6**^	**1.4 x 10**^**−3**^	**1.9 x 10**^**−6**^	**1.1 x 10**^**−6**^
3	F_1_:C57m			-	NS	NS	NS
4	F_1_:129m				-	3.0 x 10^−2^	4.2 x 10^−2^
5	129 H3i					-	NS
6	129 H3i-fh						-
**T1 sternal ribs**		C57 cb	C57 fb	F_1_:C57m	F_1_:129m	129 H_3_i	129 H_3_i-fh
1	C57 cb	-	NS	**1.1 x 10**^**−5**^	**1.5 x 10**^**−4**^	**3.3 x 10**^**−9**^	**1.1 x 10**^**−10**^
2	C57 fb		-	**2.7 x 10**^**−4**^	**3.1 x 10**^**−3**^	**8.7 x 10**^**−8**^	**2.2 x 10**^**−9**^
3	F_1_:C57m			-	NS	NS	NS
4	F_1_:129m				-	**1.6 x 10**^**−3**^	**4.2 x 10**^**−4**^
5	129 H3i					-	NS
6	129 H3i-fh						-
**T1 sternal rib attachment**		C57 cb	C57 fb	F_1_:C57m	F_1_:129m	129 H_3_i	129 H_3_i-fh
1	C57 cb	-	NS	NS	NS	**5.7 x 10**^**−8**^	**1.5 x 10**^**−8**^
2	C57 fb		-	NS	NS	**2.6 x 10**^**−8**^	**5.1 x 10**^**−9**^
3	F_1_:C57m			-	NS	**8.9 x 10**^**−5**^	**3.7 x 10**^**−5**^
4	F_1_:129m				-	**5.1 x 10**^**−8**^	**8.0 x 10**^**−9**^
5	129 H3i					-	NS
6	129 H3i-fh						-
**T2 sternal rib attachment**		C57 cb	C57 fb	F_1_:C57m	F_1_:129m	129 H_3_i	129 H_3_i-fh
1	C57 cb	-	NS	NS	NS	**1.1 x 10**^**−5**^	**3.1 x 10**^**−6**^
2	C57 fb		-	NS	NS	**2.7 x 10**^**−4**^	**4.8 x 10**^**−5**^
3	F_1_:C57m			-	NS	**6.8 x 10**^**−4**^	**4.2 x 10**^**−4**^
4	F_1_:129m				-	**1.4 x 10**^**−7**^	**4.1 x 10**^**−8**^
5	129 H3i					-	NS
6	129 H3i-fh						-

The 2-tailed Fisher’s exact test was used to evaluate significance of observed skeletal defects (uni- and bilateral combined) in each group for each feature scored. Abbreviations are the same as for Fig 5. The results are given in a matrix of pair-wise comparisons performed for each feature; p-values higher than 5.0x10^-2^ (p>0.05) were considered non-significant (NS); p-values in bold font were still significant after Bonferroni correction for multiple testing. Significant differences were found by genetic background, but not to the same degree for every feature, indicating that individual features may be controlled separately.

### Genetic background of the embryo determines manifestation of skeletal abnormalities

While the above data suggest the existence of a genetic modifier for the Hoxb6^hd^ phenotype, they do not address whether it is the genetic background of the mother or the genetic constitution of the embryo itself that determine the extent of defects in the developing skeleton. We evaluated these alternatives by crossing C57BL/6-Hoxb6^hd^ congenics to H_3_ offspring homozygous for the mutant allele. In such a cross, all animals are homozygous for the Hoxb6^hd^ allele, and resulting progeny are mixed for genetic background, with 50% contribution from C57BL/6 and 50% from a background of 87.5% 129S6/SvEv and 12.5% C57BL/6 (53.15% C57BL/6; 46.85% 129S6/SvEv). If maternal genotype contributed to phenotype severity, offspring from mothers with 129S6/SvEv contribution should exhibit the more severe phenotype, while progeny from C57BL/6-Hoxb6^hd^ mothers should exhibit a milder phenotype. This prediction was not confirmed (Fig 5, compare Columns 3 and 4), indicating that maternal strain background does not significantly affect the Hoxb6^hd^ mutant phenotype. The results instead support the conclusion that the embryonic genetic background determines the severity of the Hoxb6^hd^ mutant phenotype.

The apparent gradual increase of phenotype severity with increasing contribution from the 129S6/SvEv genetic background suggested that the effects of the Hoxb6^hd^ mutation may affect skeletal development in more quantitative than qualitative fashion. This proposition is supported by the report that simultaneous heterozygosity for mutant Hoxb6^ex1^ and Hoxb5^ex1^ alleles on opposite chromosomes causes similar defects as found in Hoxb6^ex1^ homozygotes [5]. In addition, results from compound mutants for other Hox genes provide evidence for a quantitative role of Hox protein levels in patterning of the developing limb skeleton [11]. If the overall level of Hox proteins in a given region is critical, then heterozygotes for the Hoxb6^hd^ mutation might also develop skeletal abnormalities, particularly on more susceptible genetic backgrounds, such as 129S6/SvEv. To examine this possibility, I performed post-hoc analysis of the Hoxb6^hd^ heterozygotes generated in our crosses. Fig 5 (Column 7) shows that on the C57BL/6 background, Hoxb6^hd^ heterozygotes develop normal skeletons. However, on the 129S6/SvEv background, cervical vertebral transformations and rib abnormalities were present in some Hoxb6^hd^ heterozygotes (Fig 5, Column 8). These data therefore imply that the 129S6/SvEv background confers sensitivity to the level of Hoxb6 expression. Taken together with the finding that genetic background influences phenotype penetrance in homozygotes for the Hoxb6^hd^ mutation, the data are most consistent with the conclusion that Hoxb6 function has to be viewed as a quantitative trait.

### Independent modulation of multiple skeletal features in Hoxb6 mutants by genetic background

**Fig 6:**
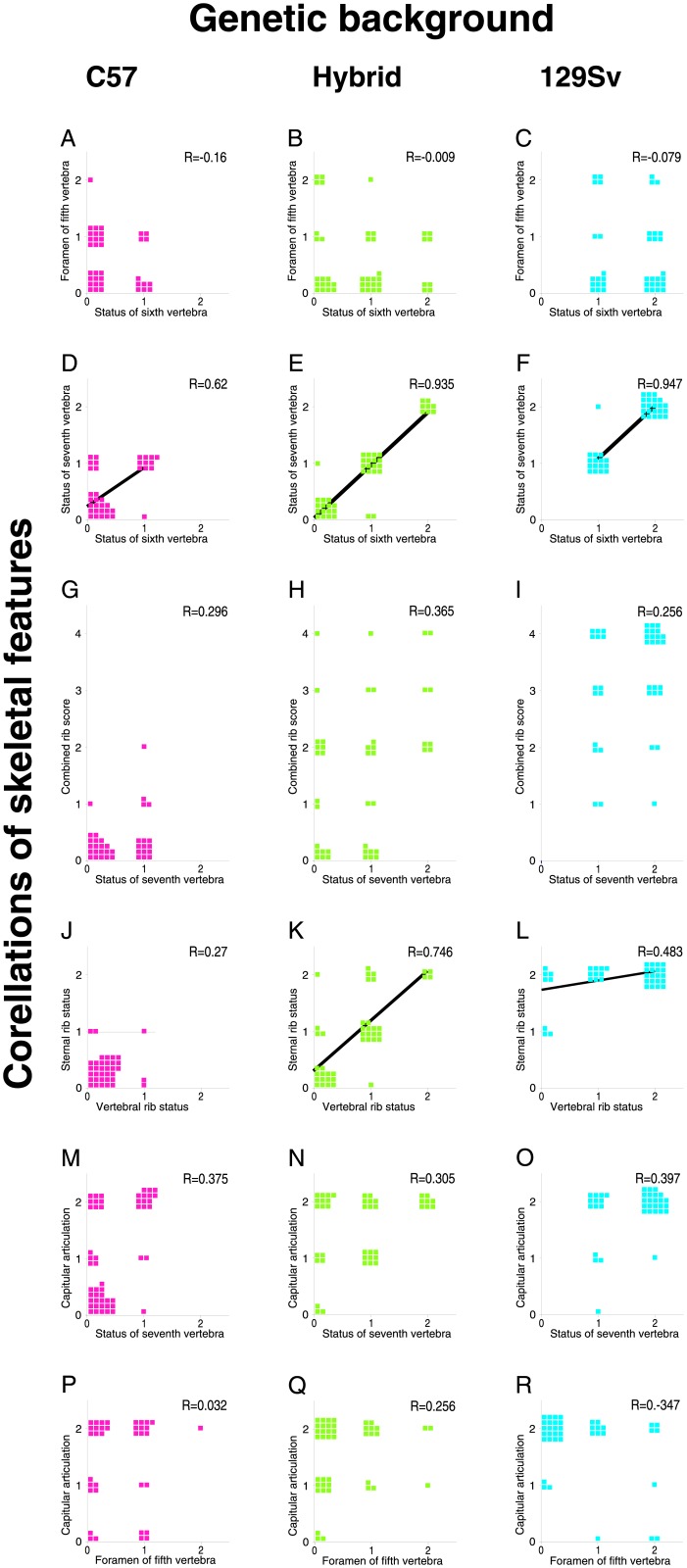
Association of skeletal features in individual Hoxb6^hd^ mutants. Regression analyses were performed using a simplified scoring scheme for each character: two points were assigned for bilateral abnormalities, one point for unilateral abnormalities and 0 for wildtype manifestation. Each animal is represented by a square. The correlation coefficients (R) were determined using Microsoft Excel. Significance for relationships was assessed by ANOVA and was smaller than p = 0.05 (adjusted p<0.003 with correction for multiple comparisons) only for data in Panels D-F, K, L (solid regression curves). The presence of association indicates that the cellular and molecular processes producing a given feature are likely developmentally linked. The absence of relationship between skeletal anomalies within individual animals indicates that the various aspects of the Hoxb6^hd^ phenotype develop independently from each other, possibly controlled by different underlying molecular mechanisms.

Interestingly, the effect of genetic background in heterozygous Hoxb6^hd^ mutants was most profound for transformations of T1->C7 (p = 0.0095). The T1->C7 vertebral transformation is also the most penetrant feature of the Hoxb6^hd^ homozygous mutant phenotype on either genetic background (see Fig 5). In order to investigate whether susceptibility to deficiency of Hoxb6 affects different skeletal elements in different ways, I analyzed whether specific features of the Hoxb6^hd^ phenotype co-occurred within individual mutant animals by grouping together the data for homozygotes on C57BL/6 background (corn-cob and non-foodstuff bedding, Fig 5 Columns 1 and 2), homozygotes on 129S6/SvEv background (H_3_ brother x sister intercrosses and matings to homozygous males, Fig 5 Columns 5 and 6) and F_1_ hybrid homozygotes (F_1_ reciprocal crosses, Fig 5 Columns 3 and 4). Due to the small number of affected animals, heterozygotes were not analyzed. For each group, pair wise associations were measured based on simplified scores for each character, giving a weight of 2 to bilateral anomalies, of 1 to unilateral anomalies and 0 to normal status. Regression analysis was performed for each group independently (Fig 6).

The character “open ventral foramen in C5” is not associated with homeotic transformations or genetic background (Fig 6, Panels A-C). Because there was also no relationship to genotype at the Hoxb6 locus, I conclude that this skeletal anomaly occurs as a natural variation in laboratory mice. These results are in agreement with natural variability of this feature observed in previous surveys of laboratory [12] and wild [13] mice.

Strong associations (Fig 6, Panels D-F) were found between vertebral transformations C6->C5 and C7->C6 on all three genetic backgrounds, indicating that while propensity for transformation is dependent on genetic background, the degree of phenotypic expression is not. Indeed, there is as strict a relationship in unilaterally affected animals as found in bilaterally affected animals (compare animals with 1:1 and 2:2 scores). These data indicate that the occurrence of C7 and C6 transformations is likely biologically coupled.

Abnormal rib development, however, was not associated with transformations of C6 or C7 (Fig 6, Panels G-I), suggesting that the effect of Hoxb6 deficiency in homeotic transformations is independent from its effect in rib development. The expression of Hoxb6 expression in ventral mesoderm at the time of sternal rib formation (see Fig 1, Panel J) is consistent with a separate role in dorsal and ventral skeletal structures. For the first rib pair, a relationship was found for vertebral (proximal) and sternal (distal) rib abnormalities, but only with contribution from the 129S6/SvEv genetic background (Fig 6, Panels K, L). The presence and absence of association between several anomalies in the Hoxb6^hd^ mutants implies that while the phenotypic manifestations are genetically linked to the Hoxb6 locus, they develop independently for the vertebral column and the ribs, respectively. The feature "capitular articulation of rib/ossified rudiment to vertebral body in T1” was independent of vertebral transformations (Fig 6, Panels M-O) or rib defects on either genetic background. This feature was also unrelated to natural variation (as represented by "open foramina in C5", Fig 5, Panels P-R). From these data, we conclude that "lack of capitular articulation", while dependent on Hoxb6-deficiency, constitutes yet another distinct aspect of the Hoxb6^hd^ phenotype, independent of homeotic transformations or rib defects.

**Fig 7:**
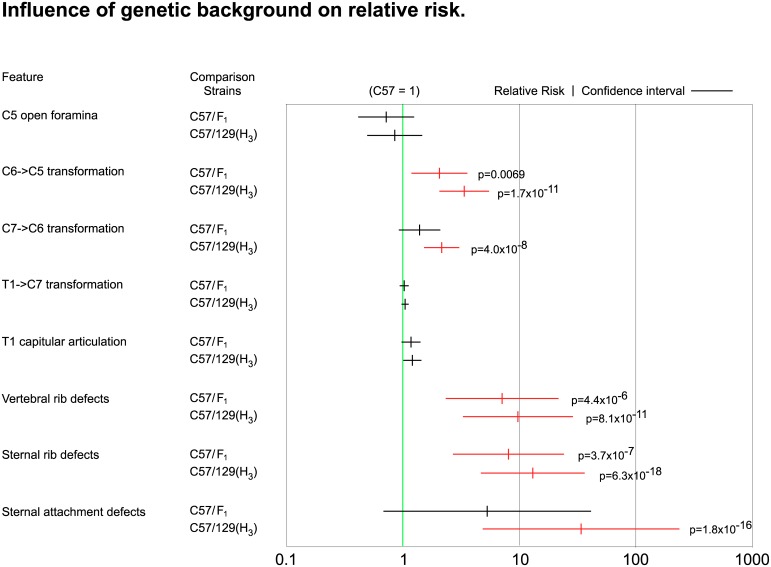
Influence of genetic background on risk for skeletal abnormalities in Hoxb6 mutants. The fraction of animals with abnormalities (uni- and bilateral combined) for each group was determined and risk was calculated relative to C57BL/6-Hoxb6^hd^ homozygous mutants. Relative Risk increases with increasing contribution from the 129S6/SvEv genetic background; for some features, this increase is significant (p<0.05) in both chi-square and Fisher's exact test (p-values are shown). Confidence intervals (horizontal bars) were determined for the comparisons of relative risk estimate (small vertical bar) for Hoxb6 mutants on F1 hybrid and 129S6/SvEv genetic background, respectively, based upon the results in S2 Table. Relative risk for anomalies in Hoxb6^hd^ mutants on C57BL/6 background was set to 1 (green line), and risk estimates whose confidence intervals do NOT include the value 1 are highly significant as indicated by red color of the data points.

The conclusion that genetic background influences individual skeletal features in different manner and independent from each other is further supported by the risk estimates for an individual to develop anomalies (Fig 7 and S2 Table). Contribution from the 129S6/SvEv genetic background increases relative risk for vertebral transformations up to 3-fold, risk for rib defects up to 12-fold, and risk for sternal attachment defects up to 32-fold, while risk for T1 transformation remains constant across genetic backgrounds. The difference in risk and rate of increase for different features strongly supports the notion of distinct, largely independent, effects of genetic background on the underlying developmental processes that give rise to the skeletal structures at the cervico-thoracic junction.

## Discussion

### Hoxb6 controls multiple independent processes in skeletal development

The skeletal phenotype of Hoxb6^hd^ mutants consists of distinct aspects that are differentially affected by genotype at the Hoxb6^hd^ mutant allele and genetic background. Ten prominent features were analyzed: "Open foramen in C5", "Transformation of C6->C5", "Transformation of C7->C6", "Transformation of T1->C7", "Articulation of ribs to the eighth vertebra", "Formation of vertebral rib on the eighth vertebra", "Formation of sternal rib on the eighth vertebra", "Rib attachment at first sternebra", "Rib attachment at second sternebra", "Open foramen in C5" and "Spinous process on the ninth vertebra" (Figs 7, 1D and 2K). The spinous process was found absent more often on the 129S6/SvEv background (Fig 1, Panel D, and Fig 2, Panel K), but since it is not fully developed at birth, this character was difficult to score in newborn skeleton preparations and therefore excluded from the quantitative analysis. “Open foramina in C5” appeared naturally variable between individuals (see above). Such idiopathic variations have been ascribed to maternal age, parity and litter size (each with up to 10% of the variance [14]), and to intractable factors within the individual (up to 80% of the variance [15]). However, the other eight skeletal features in Hoxb6^hd^ mutants were dependent on factors identified in this study: Hoxb6^hd^ mutant allele gene dosage and genetic background.

### Homeotic transformations in Hoxb6 mutants exhibit differential penetrance by axial position

Genetic background is an important parameter for penetrance of the homeotic transformations C6->C5 and C7->C6; the incidence of both is reduced on the C57BL/6 background compared to 129S6/SvEv. In the latter mutants, transformations are more often found bilaterally, but the types of shape change or severity do not differ between strains; the shapes of transformed elements in unilaterally affected individuals are indistinguishable from those found in animals where transformations occurred bilaterally. This indicates that the unit affected by Hoxb6 deficiency is the combined sclerotome for one side of the vertebra, and that incorporation of the transformed portion into the vertebral body as a whole proceeds normally. Thus, our results are consistent with the generally accepted notion that vertebral identity, as well as the cervico-thoracic and the thoracic-lumbar boundary, respectively, is determined prior to or during somite formation, and stable thereafter [16–19].

Transformations of C7->C6 were more frequent than C6->C5, on either genetic background, and even in heterozygotes (on the 129S6/SvEv background). This suggests that proximity to the fully penetrant T1->C7 transformation could influence the propensity for more rostral transformations. Alternatively, more rostral vertebrae may be less susceptible to deficiency of Hoxb6. C6 derives from the region of anterior-most expression of Hoxb6, which may not express Hoxb6 at uniform levels (see Fig 1, Panel I, red arrow). If C6 is thus composed of a mixture of cells, the overall effective concentration within the precursor pool for the entire structure could vary between animals, predisposing some to transformation to C5 identity in this segment. Combined with the graduated penetrance of the cervical homeotic transformations on different genetic backgrounds, these results are most compatible with a model where quantitative levels of Hoxb6 expression affect vertebral identity.

### Rib development in Hoxb6 mutants

A quantitative role for Hoxb6 is also supported by our analysis of rib abnormalities in the Hoxb6 mutants. Ribs associated with the eighth vertebra form either with bone and cartilage, or as ossified rudimentary stubs only (more prevalent on the 129S6/SvEv genetic background). Where first ribs are proximally absent, the second ribs often assume first rib identity, with relatively shorter bony portion and broader cartilage. Fusions of first with second ribs are also frequent, typically occurring proximal to or at the osseous-cartilaginous junction (also described by Rancourt et al. for Hoxb6^ex1^ mutants [5]), and are often accompanied by bifurcation of the second rib cartilage further distal (see Fig 2). In the chick, rib fusions and bifurcations can be caused by increased FGF8 signaling, and a decrease in FGF8 causes rib deletions [20], suggesting a possible involvement of FGF signaling in the Hoxb6 mutant phenotype. The fact that ribs can be formed despite the T1->C7 vertebral transformation indicates that skeletal precursor cells emanating from transformed sclerotome retain the capacity for migration through the lateral mesoderm, and for normal rib formation. This capacity may be reduced on the 129S6/SvEv genetic background, the migrating cells may be less responsive to signals required for rib formation, or less signal may be present. The gradual increase in defective rib formation with increasing contribution from the 129S6/SvEv genetic background again supports the hypothesis of quantitative rather than qualitative effects of Hoxb6 deficiency in rib development.

### Development of ventral skeletal structures in Hoxb6 mutants

Sternal abnormalities in Hoxb6 mutants have a strong association of with rib defects: Aberrant attachment positions for ribs from T2 and T3 are only found if ribs in T1 are completely, or at least ventrally, absent (correlation coefficient of 0.63 on the 129S6/SvEv background). In these cases, attachments at the first sternebra are either missing, or cartilage (from bifurcated T2 ribs or independently formed) attaches at some point anterior to the normal position for second ribs, resulting in ossification with off-set register for the two lateral halves of the sternebra. Ribs formed from the T3 segment in the Hoxb6^hd^ mutants either attach at the T2 position, reducing the overall number of sternebrae by one, or they attach at the normal T3 position, leaving a lengthened sternebra, consistent with fusion (non-separation) of first and second sternebra as a result of loss of rib signaling. From the most severe cases of off-set rib attachment (see Fig 2), I conclude that both sides of the sternum are patterned independently, prior to fusion of the sternal bands [21, 22], with relevant positional information imparted by the ribs [23]. Unilaterally, these signals may differ within the same segment, demonstrating that the rib and sternal progenitor cells retain competence for either positional fate. In ventral mesoderm, Hoxb6 is only expressed after somitogenesis, supporting a role for this transcription factor in processes that are distinct in time and space. The results of the present study thus show that Hoxb6 regulates multiple processes underlying pattern formation in the developing skeleton.

### Lateral variations in skeletal patterning

The occurrence of unilateral defects was noted also for another Hoxb6 mutant allele on mixed genetic background (Hoxb6^ex1^ [5]). The authors argued that, because each individual has a defined and uniform genetic composition, genetic background could not account for lateral differences. However, our results clearly show that the overall propensity for unilateral or bilateral transformations and rib abnormalities is dependent on genetic background, with relative risk for anomalies increasing with higher contribution from the 129S6/SvEv genetic background (Fig 7). Historic references debate the influence of sex on occurrence of unilateral skeletal anomalies [24, 25], but we found no significant differences between males and females in our crosses. Therefore, unilateral or bilateral anomalies result from interaction of homozygosity at the Hoxb6^hd^ mutant allele with genetic background. Where unilateral transformations are present, they all occur on the same side of the animal, although we did not observe a preference for side. Thus, positional identity is coupled between segments on the same side but laterally independent of each other. This is consistent with data on human skeletal asymmetries [24].

The most intuitive conceivable difference between one body side developing with transformations and one without would be temporal differences [26] in axial patterning on one side of the animal relative to the other. This notion implies that two parameters interact in patterning the skeletal elements in the cervico-thoracic region: Hox gene expression levels and the temporal progression of somitogenesis. Then, individual somites can be envisioned as having some way of 'measuring' time. The alternative concept that they possess an internal 'record' of position, cannot explain the occurrence of unilateral transformations, since -by definition- both sides of a vertebra have the same rostral-caudal position. Instead, I envision that temporal offset in somitogenesis produces the same number of somites on each side, keeping the register of prechondrogenic condensations, but developmental time elapsed relative to a 'set-point' is recorded and/or interpreted differently in elements that undergo transformation. Whether or not temporal asynchrony affects whole regions of the developing skeleton concurrently, or each newly formed somite sequentially, cannot be answered from the data available, although these alternatives are not mutually exclusive.

Notably, the affected structures in Hoxb6^hd^ mutants derive from the caudal half of each somite [27, 28]. It has recently been shown that different compartments of somites activate transcription of cyclically expressed genes [29] with temporal delay relative to medial-lateral orientation [30, 31], providing a potential basis for differences in record-keeping of time. A prediction from our results is that caudal-lateral somite halves are less tightly regulated, allowing for strain differences, while rostral halves may be more strictly tied to the temporal control of somitogenesis, the 'somite clock' [32]. This hypothesis is testable by investigating 'clock' genes in both strains.

### Skeletal pattern as a quantitative trait

I propose that skeletal pattern, as revealed in our Hoxb6 mutants, has to be considered as a quantitative trait. While the outcomes are apparently dichotomous (structure present vs. absent, identity normal vs. transformed), the incidence of defects and the propensity for normal or abnormal development are modulated by genetic modifiers in a quantitative fashion. The greater penetrance of skeletal anomalies in mutants on the 129S6/SvEv background suggests that this strain might be more susceptible to perturbation (by the Hoxb6^hd^ mutation), or have less capacity for repair and/or compensation than the C57BL/6 strain. However, the 129S6/SvEv genetic background does not simply increase overall variability: within-group variance was comparable (0.21/0.25 in C57BL/6, and 0.24/0.24 in 129S6/SvEv) for the homeotic transformations of C6 and C7, as well as for other features. I therefore conclude that each inbred strain contains one or more specific alleles that act as modifiers in skeletal patterning. Modifier loci in the genetic background can be fine-mapped by quantitative trait analysis, and we are currently conducting such experiments.

One implication from our results is that Hox gene mutations in general may have greater expressivity in the 129S6/SvEv strain. This has indeed been found for mutant alleles of Hoxb4 [4] where sternal defects have greater penetrance on the 129SvEv background compared to 129 x C57 hybrid background. Conversely, stronger phenotypes on the C57BL/6 background were reported for mutations of the Tlx2 [33] and Otx [34] genes, as well as for deficiencies of TGF-β [35, 36]. Thus, the modifier(s) detected here act(s) specifically on Hox-controlled phenotypes.

### Genetic modifier(s) for Hox gene function

Our results allow several inferences about the nature and specificity of the genetic modifier(s):

1) The modifier is unlikely to be the mutant Hoxb6^hd^ allele itself, which was targeted in 129Sv-derived embryonic stem cells. As the mutant locus was backcrossed to the C57BL/6 background, we expect that the entire Hoxb cluster in all of our mice is of ES cell (129Sv-Hoxb6^hd^) origin, owing to the lack of recombination, as specifically demonstrated for the Hoxb cluster [37]. Thus, cis-regulatory elements and genomic environment of the Hoxb cluster should be identical in all our Hoxb6^hd^ mutants regardless of genetic background. This excludes neighboring Hox genes on the Hoxb cluster as potential modifiers.

2) A modifier may affect Hoxb6 function by conferring general susceptibility to the 129S6/SvEv genetic background, in a quantitative manner. Here, the intermediate penetrance of the phenotype in the reciprocal F_1_ hybrid crosses is informative: occurrence of appreciable phenotype severity (including bilateral effects) in the F_1_ hybrid mutant group argues against dominant effects from a C57BL/6 modfier. Dominant action of a single modifier locus from 129S6/SvEv is also excluded: with exception of the T1->C7 transformation, the next most penetrant feature (transformation C7 -> C6) is present in only 73.8% (31/42) rather than 100% of the hybrids (see Fig 5). By analogy, recessive action of a 129S6/SvEv modifier is unlikely. Instead, the presence of some severe phenotypes in F_1_ hybrids, and the gradual increase of phenotype expressivity with larger contribution from 129S6/SvEv suggest that a general genetic modifier acts in permissive intermediate fashion. Backcrosses of F_1_ hybrid Hoxb6^hd^ homozygotes to either C57BL/6-Hoxb6^hd^ or 129S6/SvEv Hoxb6^hd^ mutants, respectively, confirming this (see Fig 4): the "missing rib" phenotype is found with penetrance intermediate between the parental populations, favoring intermediate genetic function for the modifier(s).

3) The modifier(s) has diverse influences on specific features of the phenotype (compare columns 3/4 to 5/6 in Fig 5): Higher contribution from 129S6/SvEv is required for increased penetrance of aberrant rib articulation at the sternum (see Fig 5, Panels G, H) than is required to increase penetrance of rib defects (Fig 5, Panels E, F) or cervical vertebra transformations (Fig 5, Panels A, B). The low frequency of sternal articulation defects in F_1_ hybrid mutants supports the possibility that a single allele—for which homozygosity at the 129Sv/Ev derived locus would be required—controls penetrance of the sternal articulation phenotype. By the same logic, for increased penetrance of rib or cervical vertebra transformation phenotypes, heterozygosity for (a) 129S6/SvEv derived allele(s) is sufficient; homozygosity at such a locus (loci) may augment penetrance. Thus, genetic background affects vertebral identity and rib development in intermediate, and sternal articulation in recessive fashion. The respective modifiers are postulated to act independently of each other.

Furthermore, the expressivity of different features of the Hoxb6 phenotype varies: In first rib development, severity ranges from perturbations to complete absence. In contrast, vertebral identity presents in binary states: either normal or transformed. The lack of association between rib defects and vertebral transformations within individual animals indicates that these characters are under independent control. These genetic differences may reflect the involvement of lateral mesoderm-derived signals [38] in the development of ventral skeletal structures [39]. Thus, the Hoxb6 mutant phenotype is influenced by modifier loci that act in general and region-specific fashion—and possibly independent of each other—on several aspects that contribute to skeletal patterning: time-keeping in the somite (as indicated by homeotic transformations), potency of the caudal half of the somite (as indicated by proximal rib development), migration/condensation of distal rib precursors (as indicated by ventral rib development and sternal articulation), which in turn influence patterning of the sternum. Our breeding experiments have thus revealed multiple functions for Hoxb6 in skeletal patterning that are mediated in interaction with the genetic modifier(s).

4) The possibility of normal, or at least unilaterally normal, development in Hoxb6^hd^ mutants under any condition highlights the existence of interaction between Hoxb6 allele status and genetic background even in the presence of Hoxb6 protein. Confirmation for this proposition comes from homeotic transformations in heterozygous Hoxb6^hd^ mutants- on the 129S6/SvEv genetic background. Ocurrence was most pronounced for the T1->C7 transformations, with a trend for C7->C6 and C6->C5. From these results, I conclude that different skeletal elements within the Hoxb6 controlled region show differential sensitivity to reduction of Hoxb6 expression. Complete loss of Hoxb6 produces a similar gradient in homozygotes. In fact, phenotype manifestation can be ordered from least to most susceptible: wildtype > Hoxb6+/- on C57BL/6 > Hoxb6+/- on 129S6/SvEv > Hoxb6-/- on C57BL/6 background > Hoxb6-/- on F_1_ hybrid background > Hoxb6-/- on 129S6/SvEv background. Clearly, the graded risk for abnormal development of skeletal structures relative to Hoxb6 gene dosage and genetic background is incompatible with an instructional model for the Hox code: the Hoxb6 protein does not simply provide the instructions for C7 or T1 identity, or rib development in T1. With reference to the non-allelic non-complementation phenotypes in Hoxb6+/- Hoxb5+/- compound heterozygotes [5], and to mutants deficient in all Hox6 paralogous genes [40], it has been argued that the effective concentration of Hox proteins in a given region may be more important than the level of each specific protein. Our results provide independent support for this interpretation, by demonstrating that genetic background influences the read-out of Hoxb6 gene expression and function at the phenotypic level.

### A quantitative model for Hoxb6 function

On the basis of our evidence for the quantitative nature of Hoxb6 function, I propose a quantitative model for the manifestation of vertebral identity: In this model, the effective level of Hox protein (from several Hox genes combined) defines eventual identity of a given skeletal structure. Different structures vary in their requirement for total Hox protein levels in anterior-posterior direction, consistent with sequential collinear activation of Hox gene transcription along each cluster [41]. The model makes no implicit assumption about the biochemical nature of homeodomain protein function, but accommodates either transcriptional activator or repressor, or modulator function. The contribution of Hoxb6 to each segment is different either by expression level or functional activity, which is required for proper patterning of the five skeletal elements C6, C7, T1-T3. As it has recently been shown that Hox genes act in presomitic mesoderm [29, 32, 42, 43], and in mesoderm ingression in the primitive streak [44, 45] prior to somitogenesis, temporally different requirements may further modulate the patterning process.

Strain background effects on local Hox function can then be conceptualized as differential relative contribution by Hoxb6 (larger in 129S6/SvEv, smaller in C57BL/6), different threshold levels required for normal function, greater capacity for compensation in C57BL/6, or timing differences between the strains during gastrulation and mesoderm differentiation. One or more genetic modifiers may thus be involved in each of these processes. The different structures (vertebra, ribs) in each segment may themselves have specific threshold requirements; each feature thus developing as a composite of distinct local and temporal requirements for Hox gene function.

Observing that skeletal variations similar to the types analyzed here occurred with different penetrance in various mouse strains, Grüneberg [12, 46] proposed that they are discontinuous manifestations of an underlying distribution, assumed to be continuous, and termed them 'quasi-continuous variations'. For example, he found a higher incidence of “dystopia tuberculis anteriosum caudalis” (mislocation of the anterior tuberculum from C6 to C7, often accompanied by presence of a vertebral foramen) in the X strain than in wildtype skeletons from any other strain [47]. Unfortunately, he did not report on the vertebral shape of T1 in the affected animals, which could have resembled a homeotic transformation. Grüneberg posited that whether a structure developed normally or abnormally would be dependent on whether an individual met the specific threshold level for one or more factors. In this way, an underlying distribution of genetic parameters in a population would have apparently discontinuous outcomes in individuals. Due to the low prevalence of skeletal anomalies in wildtype mice, Grüneberg was unable to detect the genetic nature of threshold control that our experiments have now revealed.

The phenotype features in Hoxb6 mutants are consistent with the postulate of spatially restricted action [48] of a limited number of independent genetic modifiers. From crosses of C57 and BalbC mice, Green [49] estimated that a minimum of three loci would be involved in specification of skeletal type at the thoraco-lumbar boundary. We now know that the Hox genes of paralogous group 10 cooperate to pattern this boundary [50], and by analogy, one or more Hox genes overlapping with Hoxb6 expression could be involved in controlling skeletal patterning at the cervico-thoracic boundary. While Hoxb cluster genes [5] would not be detected as modifiers in our system (see above), Hox genes on other clusters are obvious candidates [40]. Several other candidate modifiers are suggested by the literature: regulators of Hox gene expression levels, such as molecules of the polycomb and trithorax groups [51–53], retinoic acid signaling [54, 55], and Cdx1 as its possible mediator [56]. Molecules that interact with Hoxb6 would be expected to influence the stochiometry of Hox gene function, and thus provide a read-out of gene dosage; Pbx1 [57, 58], Histone deacetylase [59] and the CBP histone acetyltransferase [60] have been shown to affect Hox protein function. Pbx1 has also been implicated in sternal rib development and rib fusions [57], as have FGF8 [20] and GDF11 [61] signaling. Defects in proximal/vertebral rib development implicate Pax1 and Pax9 [62, 63] as possible modifiers, and pedicle development is known to require Uncx4.1 [64, 65]. Deficiencies in these molecules affect distinct regions of the skeleton, providing the potential molecular correlates for the distinct biological processes controlled by Hoxb6. In addition, a given modifier, retinoic acid, or FGF8, for example, may influence manifestation of the Hoxb6 phenotype at different stages: prior to and during somitogenesis to produce homeotic transformations, and during migration, proliferation and differentiation of chondrogenic precursors for rib development later. Further genetic and phenotypic analyses of Hoxb6^hd^ mutants, such as by quantitative trait locus mapping, will enable us to determine the identity of genetic modifiers for Hoxb6 function in skeletal patterning.

### Relevance to human skeletal variations and the evolution of vertebrate body plans

It is interesting to note here that anteriorizations at the cervico-thoracic boundary, are exceedingly rare in humans [66], although vertebral transformations have been observed in multiple families [67]. Yet, even large population-based studies [68, 69] have not uncovered a case of complete lack of the first pair of ribs, but bifurcations of ribs were observed [68, 70]. Mutations causing absence of HOXB6 in humans may affect additional tissues [71], thus potentially reducing viability or fertility, or the role of the first rib pair in thoracic physiology, such as breathing, may be under greater functional constraint in humans. Rather, it appears that posteriorizations are more compatible with survival, and familial inheritance patterns were explainable by dominance of posteriorization [67]. Since multiple skeletal elements were typically affected, it is likely that global control mechanisms may have been altered in these cases, such as possible long-range enhancers or locus control regions for Hox clusters [72]. Thus, while the underlying molecular and genetic mechanisms of skeletal patterning are likely conserved in humans and mice [28, 73, 74], functional requirements may be subject to different selection forces. Taken together, evidences from human skeletal anomalies [24, 67] support my conclusion that skeletal patterning has to be viewed as a quantitative trait [75].

It is generally believed that alterations in the developmental mechanisms investigated here form the micro-evolutionary basis for skeletal variations that could be the basis for altered body plans [76]. The skeletal variations controlled by Hoxb6 and genetic background are compatible with survival, at least of mice in an institutional setting. While it remains unclear how variability in skeletal patterning at the population level is maintained or could be selected for, it has been speculated that subthreshold variations may confer advantage to a species, namely to provide a “cushion” of evolutionary plasticity [46] under constantly changing conditions. In Drosophila and Arabidopsis [77, 78], HSP90 plays such a role in "phenotype masking", and thus becomes a "capacitor of evolution" [79]. Identifying the genetic modifiers of skeletal patterning in mice will similarly provide insight into the evolutionary substrates that control variations in vertebrate body plans and may thus be involved in the evolution of new species.

## Supporting Information

S1 Table Litter sizes of experimental groups. (DOC)

S2 Table Influence of genetic background on risk for skeletal abnormalities in Hoxb6 mutants. (DOC)
