# Supplementary material for: Developmental Patterning as a Quantitative Trait: Genetic Modulation of the Hoxb6 Mutant Skeletal Phenotype
Source: PLoS One. 2016 Jan 22;11(1):e0146019. doi: 10.1371/journal.pone.0146019 (PMC4723086; doi:10.1371/journal.pone.0146019)
Supplement: S1 Table — (DOC) [file pone.0146019.s001.doc]

| **Supplemental Table 1: Litter sizes of experimental groups** | | | | | |  |
| --- | --- | --- | --- | --- | --- | --- |
| **Experimental Group/**  Number | | **Parents (Hoxb6 genotype)**  Males Females | | **Number of litters** | Average litter size (+ standard deviation) | |
| 1 | C57 cb | C57(-/-) | C57(+/-) | 7 | 5.5 (+2.23) | |
| 2 | C57 fb | C57(-/-) | C57(+/-) | 5 | 8.4 (+1.67) | |
| 3 | F_1_:C57m | H_3_(-/-) | C57(-/-) | 2 | 7 -- | |
| 4 | F_1_:129m | H_3_(-/-) | 129(-/-) | 4 | 8.25 (+0.5) | |
| 5 | 129 H_3_i | H_3_(+/-) | H_3_(+/-) | 5 | 9.6 (+5.27) | |
| 6 | 129 H_3_i-fh | H_3_(-/-) | H_3_(+/-) | 8 | 6 (+3.33) | |
| 7 | 129 H_1_i | H_1_(+/-) | H_1_(+/-) | 6 | 8.8 (+1.72) | |

Legend to Supplemental Table 1:

Litters include heterozygotes (+/-) and homozygotes (-/-); litter sizes were not significantly different. The trend to apparently lower litter sizes in group 1 is nost statistically significant and was not confirmed in many more litters generated from this line for other experiments.
