# Supplementary material for: Developmental Patterning as a Quantitative Trait: Genetic Modulation of the Hoxb6 Mutant Skeletal Phenotype
Source: PLoS One. 2016 Jan 22;11(1):e0146019. doi: 10.1371/journal.pone.0146019 (PMC4723086; doi:10.1371/journal.pone.0146019)
Supplement: S2 Table — (DOC) [file pone.0146019.s002.doc]

**Supplemental Table 2:**

**Influence of genetic background on risk for skeletal abnormalities in Hoxb6 mutants.**

|  | Genetic background | C57 | F_1_ | 129(H_3_) |
| --- | --- | --- | --- | --- |
|  | Hoxb6 status | -/- | -/- | -/- |
|  | Total number examined | 36 | 42 | 38 |
| Feature |  |  |  |  |
| C5 open foramina | Number of animals affected | 17 | 14 | 15 |
|  | Incidence | 0.472 | 0.333 | 0.395 |
|  | Relative Risk | 1 | 0.71 | 0.836 |
|  |  |  |  |  |
| C6->C5 transformation | Number of animals affected | 11 | 26 | 38 |
|  | Incidence | 0.306 | 0.62 | 1 |
|  | Relative Risk | 1 | **2.03** | **3.27** |
|  |  |  |  |  |
| C7->C6 transformation | Number of animals affected | 17 | 27 | 38 |
|  | Incidence | 0.472 | 0.643 | 1 |
|  | Relative Risk | 1 | 1.36 | **2.5** |
|  |  |  |  |  |
| T1->C7 transformation | Number of animals affected | 35 | 41 | 38 |
|  | Incidence | 0.972 | 1 | 1 |
|  | Relative Risk | 1 | 1.029 | 1.029 |
|  |  |  |  |  |
| T1 capitular articulation | Number of animals affected | 29 | 39 | 36 |
|  | Incidence | 0.806 | 0.929 | 0.947 |
|  | Relative Risk | 1 | 1.24 | 1.18 |
|  |  |  |  |  |
| Vertebral rib defects | Number of animals affected | 3 | 24 | 30 |
|  | Incidence | 0.083 | 0.571 | 0.789 |
|  | Relative Risk | 1 | **6.85** | **9.47** |
|  |  |  |  |  |
| Sternal rib defects | Number of animals affected | 3 | 27 | 38 |
|  | Incidence | 0.083 | 0.643 | 1 |
|  | Relative Risk | 1 | **7.714** | **12.05** |
|  |  |  |  |  |
| Sternal attachment defects | Number of animals affected | 1 | 6 | 35 |
|  | Incidence | 0.028 | 0.143 | 0.921 |
|  | Relative Risk | 1 | 5.14 | **33.16** |

Legend to Supplemental Table 2:

Relative risk ratios were calculated with the incidence in C57BL/6 set to 1. Bold: Significant increase of relative risk compared to C57BL/6 (after adjustment of p-values for multiple testing).
